# Investigation of the Enteric Pathogenic Potential of Oral *Campylobacter concisus* Strains Isolated from Patients with Inflammatory Bowel Disease

**DOI:** 10.1371/journal.pone.0038217

**Published:** 2012-05-30

**Authors:** Yazan Ismail, Vikneswari Mahendran, Sophie Octavia, Andrew S. Day, Stephen M. Riordan, Michael C. Grimm, Ruiting Lan, Daniel Lemberg, Thi Anh Tuyet Tran, Li Zhang

**Affiliations:** 1 The School of Biotechnology and Biomolecular Sciences, University of New South Wales, Sydney, Australia; 2 Department of Gastroenterology, Sydney Children’s Hospital, Sydney, Australia; 3 School of Women’s and Children’s Health, University of New South Wales, Sydney, Australia; 4 Department of Paediatrics, University of Otago, Christchurch, New Zealand; 5 Gastrointestinal and Liver Unit, The Prince of Wales Hospital, Sydney, Australia; 6 St. George Clinical School, University of New South Wales, Sydney, Australia; 7 Faculty of Medicine, University of New South Wales, Sydney, Australia; Charité-University Medicine Berlin, Germany

## Abstract

**Background:**

*Campylobacter concisus*, a bacterium colonizing the human oral cavity, has been shown to be associated with inflammatory bowel disease (IBD). This study investigated if patients with IBD are colonized with specific oral *C. concisus* strains that have potential to cause enteric diseases.

**Methodology:**

Seventy oral and enteric *C. concisus* isolates obtained from eight patients with IBD and six controls were examined for housekeeping genes by multilocus sequence typing (MLST), Caco2 cell invasion by gentamicin-protection-assay, protein analysis by mass spectrometry and SDS-PAGE, and morphology by scanning electron microscopy. The whole genome sequenced *C. concisus* strain 13826 which was isolated from an individual with bloody diarrhea was included in MLST analysis.

**Principal Findings:**

MLST analysis showed that 87.5% of individuals whose *C. concisus* belonged to Cluster I had inflammatory enteric diseases (six IBD and one with bloody diarrhea), which was significantly higher than that in the remaining individuals (28.6%) (*P*<0.05). Enteric invasive *C. concisus* (EICC) oral strain was detected in 50% of patients with IBD and none of the controls. All EICC strains were in Cluster 1. The *C. concisus* strain colonizing intestinal tissues of patient No. 1 was closely related to the oral *C. concisus* strain from patient No. 6 and had gene recombination with the patient’s own oral *C. concisus.* The oral and intestinal *C. concisus* strains of patient No. 3 were the same strain. Some individuals were colonized with multiple oral *C. concisus* strains that have undergone natural recombination.

**Conclusions:**

This study provides the first evidence that patients with IBD are colonized with specific oral *C. concisus* strains, with some being EICC strains. *C. concisus* colonizing intestinal tissues of patients with IBD at least in some instances results from an endogenous colonization of the patient’s oral *C. concisus* and that *C. concisus* strains undergo natural recombination.

## Introduction

*Campylobacter oncisus* is a Gram-negative bacterium with a curved shape and a polar flagellum, which was first isolated from human gingival plaques in 1981 [1]. *C. concisus* is a fastidious bacterium, requiring hydrogen enriched microaerobic conditions for growth [2], [3].

Recently, *C. concisus* has been shown to be associated with inflammatory bowel disease (IBD). IBD is a chronic inflammatory disorder of the gastrointestinal tract; the two major forms of IBD are Crohn’s disease (CD) and ulcerative colitis (UC) [4], [5]. The aetiology of IBD is unknown. Studies have shown that multiple factors including genetic factors, environmental factors and intestinal microflora are involved in the development of IBD [4], [5]. Despite strong evidence showing that the intestinal microbiota plays a key role in the pathogenesis of IBD, the exact causative or triggering agent still remains unknown [6], [7], [8].

A significantly higher prevalence of *C. concisus* in intestinal biopsies and fecal samples of patients with IBD as compared with controls were reported by a number of research groups [9], [10], [11], [12]. Using *in vitro* cell culture models, *C. concisus* was shown to increase intestinal epithelial permeability and induce intestinal epithelial production of IL-8 and apoptosis [13], [14], [15]. Some *C. concisus* strains cultured from intestinal biopsies of patients with IBD and diarrheal stool samples were shown to be invasive to Caco2 cells [14]. The presence of bacterial virulence factors such as phospholipase A2 and a cytolethal distending toxin (CDT)-like toxin in some *C. concisus* strains has been reported [16], [17].

*C. concisus* is a commensal bacterium of the human oral cavity. Zhang *et al* isolated *C. concisus* from 75% of saliva samples obtained from healthy individuals aged 3 to 60 years old and detected *C. concisus* by PCR in 95% of these samples [18]. The prevalence of *C. concisus* in the oral cavity of patients with IBD and healthy controls was not statistically different [18]. Furthermore, this study noted some bacterial protein banding similarities between a *C. concisus* strain colonizing the oral cavity and the *C. concisus* strain colonizing the intestinal tissues of a patient with IBD and proposed that specific oral *C. concisus* strains are involved in human IBD [18].

Currently, whether patients with IBD are colonized with specific oral *C. concisus* strains is not known. It is also not clear whether oral *C. concisus* strains have enteric pathogenic potential and whether *C. concisus* colonizing intestinal tissues of a given patient with IBD results from an endogenous colonization of the patient’s own oral *C. concisus*. To investigate these issues, we compared the housekeeping genes and protein profiles of oral *C. concisus* isolated from patients with IBD and controls, as well as *C. concisus* isolated from intestinal biopsies of patients with IBD. In addition, we examined the invasiveness of oral *C. concisus* isolates to Caco2 cells and identified a number of bacterial proteins that may be important to *C. concisus* invasion of Caco2 cells.

## Results

### Analysis of Housekeeping Genes of Oral and Enteric *C. Concisus* Isolated from Patients with IBD and Controls by Multilocus Sequence Typing

Six housekeeping genes amplified from 70 *C. concisus* isolates, which were obtained from eight patients with IBD and six controls (details of these *C. concisus* isolates were described in Materials and Methods section), were analysed by multilocus sequence typing (MLST). The six housekeeping genes amplified were *aspA* (aspartase A), *glnA* (glutamine synthetase), *tkt* (transkelotase), *asd* (aspartate semialdehyde dehydrogenase), *atpA* (ATP synthase alpha subunit) and *pgi* (glucose-6-isomerase). MLST analysis was based on the sequences of six housekeeping genes with a total of 2,561 bp from each isolate analysed. The sequence types (ST) and allelic profiles of *C. concisus* isolates analysed are shown in Table 1. The polymorphic nucleotides were submitted as supplementary data (Figure S1). The criteria to define strains and variants were described in the Materials and Methods section.

**Table 1:** Sequence types (ST) and allelic profiles of *C. concisus* isolated from patients with IBD and controls.

Isolate ID	Total No. of isolate	ST	*asd*	*aspA*	*atpA*	*glnA*	*pgi*	*tkt*
**P1CDO1, P1CDO5-O12, P1CDO14-O17,** **P1CDO19-O21**	16	**1**	8	7	4	6	1	1
**P1CDO3, P1CDO18**	2	**2**	2	5	3	7	7	5
**P1CDO4**	1	**3** *	8	5	4	6	1	2
**P1CDO13**	1	**4** *	1	7	4	6	1	1
**P1CDO2**	1	**5** *	8	7	4	6	1	2
**P1CDB1(UNSWCD)**	1	6@	8	4	2	8	4	3
**P2CDO1,P2CDO4**	2	**7**	7	8	5	5	8	7
**P2CDO3,P2CDO7**	2	**8**	6	6	6	4	9	8
**P2CDO5**	1	**9** *	7	8	5	5	8	8
**P2CDO2**	1	**10** *	6	8	6	4	9	8
**P2CDO6**	1	**11** *	6	6	6	4	9	7
**P3UCO1-P3UCO10, P3UCB1-P3UCB10**	20	**12**	5	1	1	1	3	4
**P3UCLW2**	1	**13**	5	1	1	1	2	4
**P3UCLW1**	1	**14**	4	2	8	3	6	4
**H1O1-H1O9**	9	**15**	3	3	7	2	5	6
**H5O1**	1	**16**	10	18	16	9	12	10
**P5CDO1**	1	**17**	11	14	14	14	10	17
**H2O1**	1	**18**	12	16	1	3	15	4
**P6CDO1**	1	**19**	13	9	15	13	19	9
**H6O1**	1	**20**	14	12	9	16	14	16
**P8UCO1**	1	**21**	15	17	13	12	18	15
**P7UCO1**	1	**22**	16	15	11	10	11	11
**H3O1**	1	**23**	17	13	12	11	17	13
**P4CDO1**	1	**24**	18	10	16	17	13	12
**H4O1**	1	**25**	9	11	10	15	16	14
***C. concisus*** ** strain 13826**		**26**	19	19	17	18	20	18

* Recombinant or mutational variants of the patient’s own oral *C. concisus* **strains.**

@ This strain, which was isolated from a patient with CD, had asd gene identical to the patient’s own oral *C. concisus* isolates.

### MLST Analysis of Oral *C. Concisus* Isolated from Patients with IBD and Controls

Among the 21 oral *C. concisus* isolates (P1CDO1-P1CDO21) obtained from patient No. 1, five sequence types (ST1- ST5) of *C. concisus* were identified (Table 1). ST1 included 16 isolates (P1CDO1, P1CDO5-O12, P1CDO14-O17 and P1CDO19-O21). ST2 included two isolates (P1CDO3 and P1CDO18). ST3, ST4 and ST5 each contained a single isolate (P1CDO4, P1CDO13 and P1CDO2) respectively. ST1 and ST2 differed in all six housekeeping genes (showing different allelic numbers at all six housekeeping genes), representing two different strains (Table 1). ST3 (P1CDO4) had four housekeeping genes (*asd, atpA, glnA*, and *pgi*) identical to ST1, *aspA* gene identical to ST2 and *tkt* gene identical to ST5, suggesting that this isolate resulted from genomic recombination between ST1, ST2 and ST5 (Table 1). ST4 (P1CDO13) had five housekeeping genes identical to ST1 and its *asd* gene was different from the patient’s oral *C. concisus* isolates, suggesting that ST4 is a recombinant of ST1 and an unsampled *C. concisus* isolate (Table 1). ST5 had housekeeping genes identical to ST1 except for one nucleotide mutation in *tkt* gene, suggesting that ST5 is a mutational variant of ST1 (Table 1 and Figure S1). Thus, two oral strains (ST1 and ST2), two recombinant variants (ST3 and ST4) and one mutational variant (ST5) were identified from patient 1.

Five STs (ST7-ST11) were identified in oral *C. concisus* isolates obtained from patient No. 2 (Table 1). ST7 contained two isolates (P2CDO1 and P2CDO4). ST8 contained two isolates (P2CDO3 and P2CDO7). ST7 and ST8 differed at all six housekeeping genes, representing two different strains (Table 1). ST9 contained one isolate (P2CDO5), which had five housekeeping genes identical to ST7 and *tkt* gene identical to ST8, suggesting that ST9 is a recombinant of ST7 and ST8 (Table 1). ST10 contained one isolate (P2CDO2), which had five housekeeping genes identical to ST8 and *aspA* gene identical to ST7, suggesting that ST10 is also a recombinant of ST7 and ST8 (Table 1). ST11 (P2CDO6) had five housekeeping genes identical to ST8, and *tkt* gene identical to ST7, suggesting that ST11 is another recombinant of ST7 and ST8 (Table 1). Thus, two oral strains (ST7 and ST8) and three recombinant variants (ST9, ST10 and ST11) were identified from patient 2 (Table 1).

The sequences of all six housekeeping genes of the 10 oral isolates (P3UCO1-O10) of patient No. 3 were identical, suggesting that this patient was colonized with a single oral *C. concisus* strain (ST12) (Table 1).

One oral *C. concisus* isolate was available from each of the remaining four patients (patients No. 5 to No. 8). The four *C. concisus* isolates from these four patients differed at all six housekeeping genes, each representing a different strain.

The nine isolates (H1O1-O9) obtained from the healthy individual No. 1 had identical housekeeping genes, suggesting that this individual was colonized with a single oral *C. concisus* strain (ST15) (Table 1). One oral *C. concisus* isolate was available from each of the remaining five healthy controls (H2-H6). Four of these healthy controls were colonized with a different *C. concisus* strain (differing at six housekeeping genes), except for H2O1 which had *atpA* and *tkt* genes identical to *C. concisus* ST12 (Table 1).

### MLST Analysis of Enteric *C. Concisus* Isolated from Patients with IBD

The intestinal biopsy *C. concisus* isolate (P1CDB1(UNSWCD)) of patient No. 1 (designated as ST6) had five unique housekeeping genes (Table 1). However, its *asd* gene was identical to the patient’s own oral ST1, ST3 and ST5, suggesting that ST6 has acquired its *asd* gene from the patient’s own oral *C. concisus* isolates (Table 1).

The 10 intestinal biopsy *C. concisus* isolates (P3UCB1-B10) obtained from patient No. 3 had six housekeeping genes identical to that of the patient’s own oral *C. concisus* isolates, indicating that the oral and intestinal biopsy *C. concisus* isolates are the same strain (ST12) (Table 1). Two luminal-washout isolates (P3UCLW1 and P3UCLW2) were obtained from patient No. 3. P3UCLW2 (ST13) had five housekeeping genes identical to the patient’s own oral and intestinal biopsy isolates (ST12) and differed only by one base in *pgi*, suggesting that ST13 is a mutational variant of ST12 (Table 1). P3UCLW1 (ST14) had six housekeeping genes that were different from the patient’s own oral and intestinal biopsy *C. concisus* isolates, representing a different *C. concisus* strain (Table 1).

### The Genetic Relationship of Oral and Enteric *C. Concisus* Isolated from Patients with IBD and Controls

**Figure 1:**
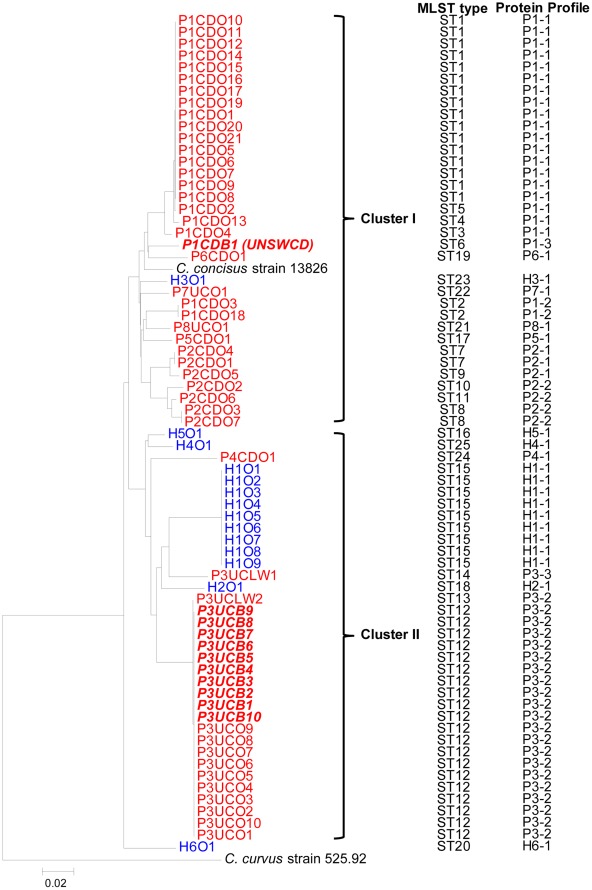
Neighbour-Joining tree based on the sequences of six housekeeping genes (*asd, aspA, atpA, glnA, pgi* and *tkt*) illustrating the phylogenetic relationships of oral and enteric *Campylobacter concisus* isolates analysed in this study. Isolates from patients with IBD are coloured red. Isolates from a healthy control are coloured blue. Clusters are indicated in roman numerals. *Campylobacter curvus* is used as an outgroup. Alongside the tree on the right are the MLST sequence types (ST) and protein profiles. *Campylobacter concisus* strain 13826 is the whole genome sequenced strain (Accession No.: CP000792.1).

To further illustrate the genetic relationship between oral and enteric *C. concisus* strains isolated from patients with IBD and controls, a phylogenetic tree was constructed based on the sequences of the six housekeeping genes analysed (Figure 1). The housekeeping genes of the whole genome sequenced *C. concisus* strain 13826 were also included.

*C. concisus* isolates obtained from the majority of the patients with IBD (6/8) formed one cluster (Cluster 1). The *C. concisus* strain (P1CDB1(UNSWCD)) isolated from intestinal biopsies of patient No. 1 with CD, *C. concisus* strain 13826 (a strain isolated from fecal sample of a patient with bloody diarrhea) and the oral *C. concisus* isolate obtained from healthy individual No. 3 were also grouped into Cluster 1. All enteric invasive *Campylobacter concisus* (EICC) strains (see results in Table 2) were grouped into Cluster 1 (Figure 1). Seven of the eight individuals (7/8, 87.5%) whose isolates belonged to Cluster 1 had inflammatory enteric diseases (six patients with IBD and one had bloody diarrhea), which was significantly higher than the remaining individuals (2/7, 28.6%) (*P*<0.05). Among the *C. concisus* isolates that did not belong to Cluster 1, six isolates were genetically closely related, forming a second cluster (Cluster 2). The oral *C. concisus* isolate obtained from a healthy control (H6O1) was genetically distinct from the other *C. concisus* isolates (Figure 1).

**Table 2:** Enteric invasive *C. concisus* (EICC) oral isolates detected in patients with IBD.

Individual ID and Clinical condition	Sample source	EICC isolates identified	Invasion Index* mean±SE
**Patient No. 1, CD**	Saliva	P1CDO3 P1CDO18	2.0±0.9 1.5±0.2
**Patient No. 1, CD**	Intestinal biopsy	P1CDB1 (UNSWCD)#	1.3±0.2
**Patient No. 2, CD**	Saliva	P2CDO1 P2CDO2 P2CDO3 P2CDO4 P2CDO5 P2CDO6 P2CDO7	9.5±0.9 6.5±1.4 11.1±3.0 6.4±1.0 5±0.9 12.3±3.2 11.2±2.8
**Patient No. 5, CD**	Saliva	P5CDO1	4.0±1.1
**Patient No. 8, UC**	Saliva	P8UCO1	3.0±0.9

* The invasion index was the average of triplicate experiments

# Positive control strain used in this study.

The intestinal biopsy isolate of patient 1 (P1CDB1(UNSWCD)) was genetically most closely related to the oral *C. concisus* isolate of a patient with CD (P6CDO1) (Figure 1). The intestinal biopsy isolates of patient No. 3 (P3UCB1-B10) were identical to the patient’s own oral isolates (P3UCO1-10). One luminal-washout isolate (P3UCLW2) of patient No. 3 was very closely related to the patient’s own oral and intestinal biopsy isolates. The second luminal-washout *C. concisus* isolate (P3UCLW1) of this patient was different from the patient oral and intestinal *C. concisus* isolates, but was more closely related to the oral isolates of healthy control No. 1 (Figure 1).

### Comparison of Protein Profiles of Different *C. Concisus* Isolates

**Figure 2:**
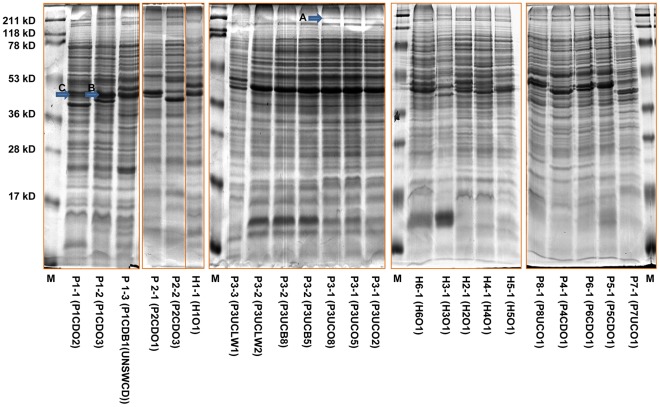
Representative whole cell protein profiles of oral and enteric *C. concisus* isolates obtained from patients with IBD and healthy controls. Arrows indicate protein bands that have been sequenced for protein identification. M: molecular weight Marker. Each lane was labelled as Protein profile (Isolate ID).

All 70 *C. concisus* isolates analyzed by MLST were also subjected to whole cell protein profile analysis. The protein profile types of all 70 *C. concisus* isolates were shown in Figure 1 and the representative protein profiles of *C. concisus* isolates from each individual are shown in Figure 2. The 21 oral *C. concisus* isolates (P1CDO1-O21) of patient 1 showed two different protein profiles (P1-1 and P1-2). Thus, the different strains showed different protein profiles, the variants did not generate new protein profiles (see MLST data). The intestinal biopsy isolate (P1CDB1(UNSWCD)) of patient No. 1 showed a protein profile (P1-3) that was different from the patient’s oral strains. The seven oral *C. concisus* isolates (P2CDO1-O7) from patient No. 2 revealed two protein profiles (P2-1 and P2-2). Again, individual strains showed different protein profiles, the recombinant variants did not generate new protein profiles. The oral *C. concisus* isolates of patient No. 3 (P3UCO1-O10) showed an identical protein profile (P3-1), consistent with the finding that all oral *C. concisus* isolates of this patient had identical sequences of housekeeping genes and representing a single strain. The intestinal biopsy *C. concisus* isolates (P3UCB1-B10) and one luminal-washout isolate of patient 3 (P3UCLW2) showed the same protein profile (P3-2), which was identical to the protein pattern of the patient’s oral *C*. *concisus* isolates except for the disappearance of a 210 kD band (band A in Figure 2). The second luminal-washout *C. concisus* isolate (P3UCLW1) showed a different protein profile (P3-3) (Figures 1 and 2).

Sequencing of the 210 kD band of P3UCO5 (oral isolate) identified 47 *C. concisus* proteins, the majority of which were ribosomal proteins and various proteins involved in metabolism. Interestingly, one protein was a bacterial virulence protein S-layer-RTX.

All oral *C. concisus* isolates of the healthy control No. 1 showed an identical *C. concisus* protein profile (H1-1) (Figure 2), consistent with the finding that all oral *C. concisus* isolates from this individual had identical housekeeping genes.

The *C. concisus* isolates obtained from the remaining patients and controls showed individual protein patterns (Figure 2).

Disease associated protein profiles were not identified.

### Detection of Enteric Invasive *C. Concisus* (EICC) Oral Strains in Patients with IBD

The invasiveness of all 70 *C. concisus* isolates to Caco2 cells was examined and expressed as invasive index. EICC strains, which have an invasive index ≥1, are shown in Table 2. EICC oral strains were detected in 50% (4/8) patients with IBD and none of the controls (0/6) (Table 2). However, the prevalence of EICC oral strains in patients with IBD was not statistically different from that in controls (*P*>0.05). The remaining *C. concisus* isolates had an invasive index <1. The positive control invasive *C. concisus* strain (P1CDB1(UNSWCD)) showed an invasion index of 1.3.

### Identification of Bacterial Proteins that may be Associated with *C. Concisus* Enteric Epithelial Invasion

Given that patient 1 was colonized with both EICC and non-EICC oral *C. concisus* isolates, we sequenced the most abundantly expressed protein band (band B shown in Figure 2) of an EICC isolate (P1CDO3) and its corresponding band (band C shown in Figure 2) of a non-EICC oral isolate (P1CDO2), attempting to identify some bacterial proteins that may be associated with *C. concisus* invasion to intestinal epithelial cells.

Twenty-three and 21 *C. concisus* proteins were identified from the protein band of the EICC isolate and the non-EICC isolate respectively. Seventeen proteins were common proteins identified from both the EICC isolate and the non-EICC isolate, and these proteins are involved in protein transport, metabolism and protein synthesis. General glycosylation pathway protein and Type II secretion system protein E, which were previously shown to be associated with bacterial virulence, were identified only from the protein band of EICC isolate. The distinctive proteins identified from the EICC isolate and the non-EICC isolate are listed in Table 3.

**Table 3:** Distinctive proteins identified from the most abundantly expressed protein band# of an oral EICC isolate and the corresponding band of a non-EICC oral *C. concisus* isolate obtained from patient 1.

EICC isolate (P1CDO3)	Non-EICC isolate (P1CDO2)
General glycosylation pathway protein* Pyridoxal phosphate-dependent enzyme Outer membrane protein Peptide chain release factor 2 Hypothetical protein CCC13826_1624 Type II secretion system protein E*	3-isopropylmalate dehydratase large subunit Glutamate dehydrogenase Signal recognition particle protein Threonine dehydratase

# Protein bands were shown in Figure 3.

* Proteins related to bacterial virulence.

### Bacterial Morphology of EICC and Non-EICC *C. Concisus* Isolates

**Figure 3:**
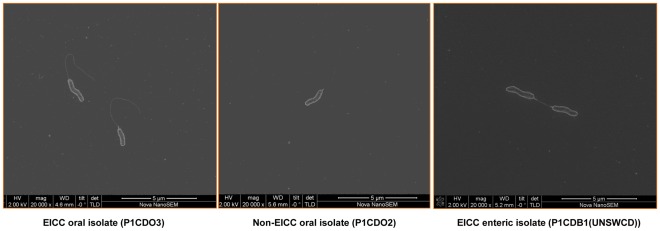
Electron Microscopic image of an oral EICC isolate, an oral non-EICC isolate and an enteric EICC isolate. All isolates were cultured from Patient No. 1.

To observe whether EICC isolates and non-EICC isolates are morphologically different, an EICC oral isolate (P1CDO3), a non-EICC oral isolate (P1CDO2) and an EICC enteric isolate (P1CDB1(UNSWCD)) were examined using electron microscopy. Both EICC and non-EICC isolates showed a similar morphology. Flagellum was present in all isolates (Figure 3).

## Discussion

This study examined whether patients with IBD are colonized with specific oral *C. concisus* strains and whether oral *C. concisus* strains have enteric pathogenic potential. Furthermore, this study investigated whether *C. concisus* colonizing intestinal tissues of a given patient with IBD results from the endogenous colonization of the patient’s own oral *C. concisus* strain.

MLST analysis revealed an association between Cluster I *C. concisus* strains and enteric inflammatory diseases including IBD (see results section). The oral *C. concisus* strains isolated from patients with IBD were predominantly grouped into Cluster I (Figure 1). EICC oral *C. concisus* strains were detected in 50% of patients with IBD and none of the controls (Table 2). All EICC oral *C. concisus* strains were in Cluster I. *C. concisus* colonizing intestinal tissues of patients with IBD, at least in some instances, originated from the colonization of the patients own oral *C. concisus* strain (Figure 1 and Table 1). Taken together, these results suggest that patients with IBD are colonized with a specific group of oral *C. concisus* strains that have enteric pathogenic potential if colonizing the intestinal tract.

Patient No. 1 was colonized with both EICC strain and non-EICC oral strains, which were morphologically indistinguishable (Figure 3). We attempted to identify proteins that may be associated with *C. concisus* intestinal epithelial invasion by sequencing the most abundantly expressed protein band of an EICC isolate and its corresponding protein band of a non-EICC isolate (Table 3 and Figure 2). Use of EICC and non-EICC *C. concisus* strains isolated from the oral cavity of the same individual would minimize the influence of environmental factors on bacterial protein expressions. We found that two of the proteins that were identified from the EICC isolate, including general glycosylation pathway protein and type II secretion system protein E, are particularly interesting in relation to bacterial virulence.

General glycosylation system has been reported to be important for *C. jejuni* to attach to and invade human epithelial cells [19]. The Type II secretion system (T2SS), consisting of at least 12 core components including T2SS protein E, is one of the five protein secretion systems that Gram-negative bacteria use to export proteins from within the bacterial cell to the extracellular environment or into host cells [20], [21]. T2SS has been shown to be important in bacterial pathogenesis [22]. A study by Iwobi *et al* showed that a novel type II secretion gene cluster is present only in high-pathogenicity *Yersinia enterocolitica* strains [23]. In our study, the fact that General glycosylation system protein and T2SS protein E were identified only from the protein band of the EICC isolate, not from the corresponding protein band of the non-EICC isolate indicates a difference of these proteins in EICC and non-EICC isolates. Future studies examining genes coding for these proteins in EICC and non-EICC isolates will further illustrate whether General glycosylation system and T2SS play a role in *C. concisus* invasion to intestinal epithelial cells.

A recent study by Kaakoush *et al* detected exotoxin 9 gene which is located in the plasmid in four enteric *C. concisus* strains that were invasive to Caco2 cells including the UNSWCD strain [24]. The presence of plasmid and the exotoxin 9 gene in oral *C. concisus* strains isolated from patients with IBD and controls should be examined in future studies. There are other evidence supporting that specific oral *C. concisus* strains may be enteric pathogenic. For example, Nielsen *et al* showed that both oral and fecal *C. concisus* strains induced epithelial barrier dysfunction [13].

The oral cavity is the primary colonization site of *C. concisus* in humans. Using a filtration method, we previously isolated *C. concisus* from 75% of saliva samples collected from healthy individuals and 85% of saliva collected from patients with IBD [18]. In comparison to the human oral cavity, the human intestine represents a less favourable environment for *C. concisus* growth. Using the same filtration method, Engberg *et al* isolated *C. concisus* from 2.8% of fecal samples collected from healthy individuals [25]. *C. concisus* requires H_2_-enriched microaerobic condition for growth [3]. H_2_ in the human intestine is generated by bacterial fermentation of unabsorbed carbohydrates; the amount of H_2_ available in an individual’s intestine is influenced by the ingested food and the composition of the local intestinal microbial community [26], [27]. Thus, whether oral *C. concisus* strains are able to establish intestinal colonization and multiply to a sufficient number to cause enteric disorders will be determined by both the properties of *C. concisus* strains and the local intestinal environment, the latter may fluctuate due to the change of ingested food and the composition of an individual’s intestinal microbial community.

Humans were previously considered as the only host of *C. concisus*, however, recently *C. concisus* was detected in fecal and saliva samples of domestic dogs and cats [28], [29]. Whether domestic pets are an additional source of human intestinal *C. concisus* infection needs to be investigated. A study by Lynch *et al* isolated *C. concisus* from chicken and beef meat [30]. While chicken and beef meat may serve as a source of human infection, it is unable to conclude whether chicken and cattle are a natural host of *C. concisus*.

Oral and intestinal biopsy *C. concisus* isolates of patient 3 revealed identical protein profiles, except for the disappearance of a 210 kD protein band in the intestinal biopsy *C. concisus* isolates (Figure 2). Interestingly, S-layer-RTX protein was identified from this 210 kD protein band of the oral isolate. S-layer is a cell surface protein found in a range of bacterial species. RTX proteins are a family of proteins secreted by a variety of Gram-negative bacteria, which exhibit various biological functions including formation of the S-layer protein in some bacterial species [31]. S-layer contributes to bacterial pathogenesis by adhering to host cells and immune evasion [32]. *Campylobacter fetus* and *Campylobacter rectus* possess S-layer [33]. High frequency antigenic variation of S-layer in *C. fetus*, resulting from DNA inversion, has been reported [33], [34]. Future studies are required to examine whether the disappearance of S-layer-RTX protein in intestinal biopsy *C. concisus* isolates in this patient is due to antigenic variation. S-layer RTX protein was previously detected in the P1CDB1(UNSWCD) strain [35]. Kalischuk *et al* reported that the gene coding for S-layer RTX was detected in two *C. concisus* strains isolated from fecal samples of healthy individuals, but not in *C. concisus* strains isolated from fecal samples of patients with diarrhea [15].

This study showed that the genome of *C. concisus* species is highly diverse. All individuals included in this study were colonized with different *C. concisus* strains, demonstrated by the findings that these *C. concisus* isolates differed at all six housekeeping genes (except for H2O1 strain, which had *atpA* and *tkt* genes identical to that of ST12) and showed unique protein profiles. This is consistent with the finding by Aabenhus *et al* [36]. Aabenhus *et al* examined 62 *C. concisus* strains using amplified fragment length polymorphism (AFLP) and found that all *C. concisus* strains gave unique AFLP profiles [36]. Our finding that *C. concisus* strains undergo natural recombination (Table 1) offer an explanation for the high genetic diversity of *C. concisus* species, as observed by other research groups [36], [37], [38], [39].

From the luminal-washout of patient No. 3, two *C. concisus* isolates were isolated. While one isolate (P3UCLW2) was the variant of the *C. concisus* strain colonizing the intestinal tissues of this patient, the other isolate (P3UCLW1) was a different strain which was closely related to the oral *C. concisus* strain of healthy control No. 1 (Table 1). This suggests that *C. concisus* detected in fecal samples may contain both *C. concisus* strains colonizing the intestinal tissues and *C. concisus* strains transiently colonizing the fecal materials.

In summary, this study provides the first evidence that patients with IBD are colonized with a group of specific oral *C. concisus* strains that may have enteric pathogenic potential. In addition, this study showed that *C. concisus*’ colonizing intestinal tissues of patients with IBD, at least in some instances, results from an endogenous colonization of the patients own oral *C. concisus* and that *C. concisus* strains undergo natural recombination.

## Materials and Methods

### Ethics Statement

’Saliva samples were obtained at the Prince of Wales Hospital, the St George Hospital and Sydney Childrens Hospital, Sydney, Australia. Ethics approvals for this study were granted by the Ethics Committees of the University of New South Wales and the South East Sydney Area Healthy Service (Ethics Nos: HREC 09237/SESIAHS 09/078, HREC08335/SESIAHS(CHN)07/48 and HREC 06233/SESAHS (ES) 06/164). Written informed consent was obtained from all subjects in this study.

### *C. Concisus* Isolates and Cultivation

A total of 70 *C. concisus* isolates obtained from eight patients with IBD and six controls were included in this study. Enteric *C. concisus* refers to isolates cultured from intestinal biopsies or luminal-washout fluid. Oral *C. concisus* refers to isolates cultured from saliva. Luminal-washout fluid was the fluid collected from luminal fluid draining tube prior to the start of the colonoscopy, which contains fecal bacteria and the mucosa associated bacteria flushed out from the intestinal mucus due to the severe diarrhoea induced during the preparation for colonoscopy. The enteric *C. concisus* isolate of patient 1 (P1CDB1) was isolated by Zhang *et al* [9], which was named as UNSWCD in a following study by Man *et al* [14]. To maintain the consistency with the previous publications and the naming system in this study, we used P1CDB1 (UNSWCD) to label this strain in this study. The enteric *C. concisus* isolates of patient 3 were isolated by Mahendran *et al* [11]. The oral *C. concisus* isolates were either isolated in our previous studies or in this study [18]. The identities of these *C. concisus* isolates were confirmed by microscopic examination of bacterial morphology and *C. concisus* specific PCR [12]. Details of the *C. concisus* isolates used in this study are listed in Table 4.

**Table 4:** *C. concisus* isolates used in this study.

Isolate ID	Number of isolates#	Sample source*	Individual ID and diagnosis
**P1CDO1-P1CDO21**	21	Saliva	Patient No. 1, CD
**P1CDB1(UNSWCD)**	1	Intestinal biopsies	Patient No. 1, CD
**P2CDO1-P2CDO7**	7	Saliva	Patient No. 2, CD
**P3UCO1-P3UCO10**	10	Saliva	Patient No. 3, UC
**P3UCB1-P3UCB10**	10	Intestinal biopsies	Patient No. 3, UC
**P3UCLW1-P3UCLW2**	2	Luminal-washout fluid	Patient No. 3, UC
**P4CDO1**	1	Saliva	Patient No. 4, CD
**P5UCO1**	1	Saliva	Patient No. 5, UC
**P6CDO1**	1	Saliva	Patient No. 6, CD
**P7UCO1**	1	Saliva	Patient No. 7, UC
**P8UCO1**	1	Saliva	Patient No. 8, UC
**H1O1-H1O9**	9	Saliva	Healthy individual No. 1
**H2O1**	1	Saliva	Healthy individual No. 2
**H3O1**	1	Saliva	Healthy individual No. 3
**H4O1**	1	Saliva	Healthy individual No. 4
**H5O1**	1	Saliva	Healthy individual No. 5
**H6O1**	1	Saliva	Healthy individual No. 6

# A total of 70 *C. concisus* isolates were examined in this study.

* *C. concisus* isolated from intestinal biopsies and luminal-washout fluid was referred as enteric *C. concisus* and *C. concisus* isolated from saliva was referred as oral *C. concisus*.

*C. concisus*%µ° isolates were cultured on Horse blood agar (HBA), prepared using Blood Agar Base No. 2 supplemented with 6 (v/v) defribinated horse blood and 10 g/ml vancomycin (Oxoid Australia Pty Limited, South Australia). Culture plates were incubated at 37C for 48 hours under microaerobic conditions generated using a *Campylobacter* gas generating kit (Oxoid).

The clinical information of patients included in this study is shown in Table 5.

**Table 5:** Clinical information of patients included in this study.

Patient ID-Sex- Age at diagnosis*	Diagnosis and disease activity at the timeof sample collection	Montreal classification [50], [51], [52]
**Patient No. 1-F-2y**	CD, new case, active	L2, L4
**Patient No. 2-M-19y**	CD, relapse, active	L3, L4
**Patient No. 3-M-23y**	UC, new case, active	Extensive E3/S1
**Patient No. 4-M-16y**	CD, remission	L3,L4
**Patient No. 5-M-13y**	CD, remission	L2,L4
**Patient No. 6-M-13y**	CD, remission	L3, L4
**Patient No. 7-M-65y**	UC, new case, active	Left sided E2/S2
**Patient No. 8-M-16y**	UC, remission	Left sided E2/S1

* *C. concisus* strains were previously isolated from biopsies of patients No. 1 and No. 3 [9], [11]. The intestinal biopsies collected from patients No. 1 and No. 3 were from caecum and descending colon respectively, sampled from areas next to inflamed mucosa. *C. concisus* was detected in intestinal biopsies collected from patients No. 2 and No. 7 by PCR [11]. No intestinal biopsies were available from patients in remission.

Patients No. 2, No. 5, No. 6 and No. 8 were being treated with anti-inflammatory drugs (infliximab, aminosalicylates, methotrexate or azathioprine) at the time of saliva collection. Patient No. 4 had ileocolonic resection and antibiotics treatment (metronidazone and ciprofloxacin) two years ago. None of the patients were receiving antibiotics treatment at the time of sample collection for this study.

### Bacterial DNA Extraction

*C. concisus*’ DNA was extracted using the Puregene DNA Extraction kit (Gentra, Minneapolis, USA) following the manufacturers instructions.

### Comparison of the Sequences of Housekeeping Genes of Oral and Enteric *C. Concisus* Isolates by Multilocus Sequence Typing

#### Choice of housekeeping genes and primer design

Six housekeeping genes (*aspA, glnA, tkt, asd, atpA and pgi)* were selected for MLST analysis. These housekeeping genes have previously been used for MLST analysis of *C. jejuni* [40], [41], [42].

The polymerase chain reaction (PCR) primers used to amplify each of the above genes were designed using the software Primer 3 plus, based on the genome sequence of *C. concisus* strain 13826 (Accession No. CP000792.1). The sequences of the PCR primers used are shown in Table 6.

**Table 6:** PCR primers used for amplification of MLST genes in this study.

Gene	′–′Forward sequence (53)	′–Reverse sequence (53)
*aspA*	ACCATGCTCGGATTTAGC	CCATCCAAACGATCACAC
*glnA*	ATGAAGCCAGAAGCGACATC	GCGTTCTCTGATCTCATCTAGG
*Tkt*	CACCGATACCTTGCTCAG	GGACACGACTACAACCAG
*Asd*	TAGAGTTATGGAGGAGGTTG	AGATAGTAGGCATCGGATAC
*atpA*	GCGGTATCACAGAAGGAAG	GCGGAGAATATGGAGGTTG
*Pgi*	GCAAGCAGCGAGGTCATC	TAGTGGCGTAGTGGTAGGC

PCR primers were designed based on the whole genome sequenced *C. concisus* strain 13826 (Accession No: CP000792.1).

#### Amplification and sequencing of MLST genes

µ×To amplify the MLST genes, hot start PCR reactions were performed in a 25 l reaction mixture containing 1 PCR buffer, 200 nM of deoxynucleotide triphosphate, 2.5 mM MgCl_2_, 5.5 U of Taq polymerase (Fisher Biotech, Subiaco, Australia), 10 pmol of each primer and 10 ng of bacterial DNA extracted from each *C. concisus*°°°° isolates. The thermal cycling conditions consist of denaturing at 96C for 2 minutes, followed by 40 cycles of 94C for 10 seconds, annealing for 10 seconds and 72C for 45 seconds. The annealing temperatures were 55C for *aspA* and *atpA*°, 51C for *tkt* and *asd*. The annealing temperatures for *gln* and *pgi*°–°™ were 55C57C, depending on individual isolates. Both strands of all PCR products were sequenced using BigDye terminator chemistry (Applied Biosystems, Foster City, CA) and separated on an ABI Capillary DNA Sequencer ABI3730 (Applied Biosystems).

#### MLST analysis

Sequences of housekeeping genes of *C. concisus* isolates were aligned and compared using software programs of MEGA 4 [43] and an in-house script MULTICOMP [44]. PHYLIP was used to generate neighbour-joining trees [45]. *Campylobacter curvus* (Accession No. CP000767.1) was used as an outgroup.

For each housekeeping gene, the different sequences present in different isolates were assigned distinct allele numbers. The allele numbers at each of the housekeeping genes defined the allelic profiles. Each isolate with a distinct allelic profile was referred as an individual sequence type (ST). Isolates with identical sequences at all six housekeeping genes were defined as the same strain. Isolates with different sequences at all six housekeeping genes were defined as different strains. In the case that a given individual was colonized with multiple *C. concisus* strains (more than one *C. concisus* strain), if evidence suggesting that some *C. concisus* isolates were generated due to gene recombination or mutations of the *C. concisus* strains colonizing the same individual, the generated *C. concisus* isolates were defined as recombinant or mutational variants.

### GenBank Sequence Submission

Sequences of housekeeping genes amplified by PCR were submitted to GenBank. The accession numbers of the sequences of the PCR products of housekeeping genes submitted to GenBank were JQ683402-JQ683505.

### Comparison of Whole Cell Protein Profiles of Oral and Enteric *C. Concisus* Isolates

Whole cell proteins of *C. concisus* isolates were prepared. Briefly, *C. concisus*µ%™µ% was harvested from HBA plates following cultivation for two days. After washing three times with PBS, the pellet was frozen-thawed three times using liquid nitrogen, then suspended in 600 l of PBS. The bacterial mixtures were sonicated on ice for 3 minutes with 0.5 seconds intervals (40 amplitudes). The protein concentrations were determined using the BCA protein assay kit (Pierce, Rockford, USA). 15 g whole cell proteins of each isolate were loaded on to 12 sodium dodecyl sulphate polyacrylamide gel electrophoresis (SDS-PAGE) to examine the whole cell protein profiles as described previously [46].

### Investigation of Enteric Invasiveness of *C. Concisus* Isolates

Invasive abilities of *C. concisus* isolates to Caco2 cells were examined using previously described gentamicin protection assay with modifications [14], .

%µ×Briefly, Minimum Essential Medium (MEM) supplemented with 10 heat inactivated fetal bovine serum (HI-FBS), 1 mM Sodium pyruvate, 0.1 mM non-essential amino acids, 100 Unit/ml Penicillin, 100 g/ml streptomycin and 225 mg/l Sodium Bicarbonate (Invitrogen Australia Pty Limited, Mulgrave, Australia) was used for routine maintenance of Caco2 cells. For gentamicin protection assay, Caco-2 cells (510^5^°% cells/well) were seeded onto 24-well cell culture plates pre-treated with rat tail collagen type 1 (0.05 mg/ml) (BD Australia, North Ryde, Australia) and incubated at 37C with 5 CO_2_ for 4 days to form a monolayer. The monolayer was washed 4 times using Phosphate Buffer Saline (PBS) and incubated with *C. concisus* isolates at a multiplicity of infection (MOI) of 100 for 2 hours in MEM media containing no antibiotics. Six wells of Caco2 cells were infected with each *C. concisus*% isolate. Following the 2 hour incubation, the wells were washed 4 times with PBS. The Caco2 monolayer of three wells was lysed with 1 Triton X-100 for 5 minutes. Serial dilutions of cell lysates were inoculated onto HBA plates and incubated as described above. Colony-forming units (CFU) of *C. concisus* were recorded, which were regarded as the numbers of *C. concisus* that have adhered to Caco2 cells. The remaining three wells of Caco2 cells infected with *C. concisus*µ% were incubated with 1 ml of MEM containing 200 g/ml of gentamicin to kill the extracellular bacteria. Following washing of the Caco2 monolayer for 5 times using PBS, the cells were lysed with 1 Triton X-100. Serial dilutions of cell lysates were inoculated onto HBA plates and incubated for two days. CFU of *C. concisus* were recorded, which were regarded as the numbers of *C. concisus* that have been internalised into Caco2. Gentamicin assay was repeated three times.

Invasive index, which is the number of internalized *C. concisus/*number of adherent *C. concisus*× 100, was calculated using the formula described by Larson *et al* [49]. A *C. concisus*≥ strain that has an invasive index 1 was defined as an enteric-invasive *C. concisus* (EICC) strain. This definition was based on previous studies showing that the established human enteric pathogen *Campylobacter jejuni*≥ with an invasive index 1 causes symptoms in infected pigs similar to human symptoms [49]_._[ The UNSWCD strain, which was previously shown to invasive to Caco2 cells 14), was used as the positive control.

### Mass Spectrometry Analysis

A number of *C. concisus* protein bands separated on SDS-PAGE were subjected to mass spectrometry analysis to identify proteins that may be important for *C. concisus* invasion to Caco2 cells and for *C. concisus* intestinal colonization. Briefly, protein bands of interest were excised from Commassie Blue stained polyacrylamide gel and were digested with trypsin. Digested peptides were separated by nano-liquid chromatography (LC) using an Ultimate 3000 HPLC and autosampler system (Dionex, Amsterdam, The Netherlands) and then subjected to analysis using a LTQ-FT Ultra mass spectrometer (Thermo Electron, Bremen, Germany). All MS/MS spectra were searched against NCBI database using MASCOT (version 2.3). Mass spectrometric analysis was carried out at the Bioanalytical Mass Spectrometry Facility, University of New South Wales, Australia.

### Scanning Electron Microscopy

Scanning electron microscopy was used to examine the morphology of two different oral *C. concisus*>< isolates, one shown to have an invasive index 1 (defined as EICC in this study) and another shown to have an invasive index 1 (defined as non-EICC in this study). *C. concisus*%°% suspension prepared using MEM containing 10 HI-FBS was placed on glass cover slips. The glass cover slips were incubated at 37C with 5 CO_2_%% for 2 hours, the bacteria were fixed with 2 glutaraldehyde and 2.5 paraformadehyde in 0.1 mol/L phosphate buffer (pH 7.4). Following dehydration in ethanol and critical point drying, *C. concisus*™ was mounted on carbon tabs, gold-coated then observed using Nova NanoSEM 230 high resolution scanning electron microscope (FEI, Oregon, USA). Scanning electron microscopic examination was performed at the Electron Microscope Unit at the University of New South Wales, Australia.

### Statistic Analysis

’Fishers exact test (two tailed) was used in this study. Statistical analysis was performed using GraphPad Prism 5 software (San Diego, CA).

## Supporting Information

Figure S1 **The polymorphic nucleotides of MLST genes and Sequence types (ST) of** ***C. concisus*** **isolates.** Six housekeeping genes (*asd, aspA, atpA, glnA, pgi* and *tkt*) of 70 oral and enteric C. concisus* isolates cultured from eight patients with IBD and six healthy control were analysed. Dot indicates that the base is the same as the consensus. The whole genome sequenced *Campylobacter concisus* strain 13826 (Accession No.: CP000792.1).
(DOCX)
