# Supplementary material for: Investigation of the Enteric Pathogenic Potential of Oral Campylobacter concisus Strains Isolated from Patients with Inflammatory Bowel Disease
Source: PLoS One. 2012 May 30;7(5):e38217. doi: 10.1371/journal.pone.0038217 (PMC3364211; doi:10.1371/journal.pone.0038217)
Supplement: Figure S1 — The polymorphic nucleotides of MLST genes and Sequence types (ST) of C. concisus isolates. Six housekeeping genes (asd, aspA, atpA, glnA, pgi and tkt) of 70 oral and enteric C. concisus* isolates cultured from eight patients with IBD and six healthy control were analysed. Dot indicates that the base is the same as the consensus. The whole genome sequenced Campylobacter concisus strain 13826 (Accession No.: CP000792.1). (DOCX) [file pone.0038217.s001.docx]

Figure S1

**MLST | *asd*.................. .| *aspA***

**type Consensus TACCTGCTTTCAGTCTCCCGTGTGTTGTTTACCCGTATGACCAACCGAACCGTCGCTCTCCCACACGTAGTAGGAATATGTCACGAATGAGCGGCCTTGAGGTGGGGTCCACGTCAGATG**

1 P1CDO10 C......CCC.G...CTT.......C....T...........T...T.C...C......T...TGT.G.......G.....GG..........A..C...A.....AA...ATA.....A

1 P1CDO11 C......CCC.G...CTT.......C....T...........T...T.C...C......T...TGT.G.......G.....GG..........A..C...A.....AA...ATA.....A

1 P1CDO12 C......CCC.G...CTT.......C....T...........T...T.C...C......T...TGT.G.......G.....GG..........A..C...A.....AA...ATA.....A

1 P1CDO14 C......CCC.G...CTT.......C....T...........T...T.C...C......T...TGT.G.......G.....GG..........A..C...A.....AA...ATA.....A

1 P1CDO15 C......CCC.G...CTT.......C....T...........T...T.C...C......T...TGT.G.......G.....GG..........A..C...A.....AA...ATA.....A

1 P1CDO16 C......CCC.G...CTT.......C....T...........T...T.C...C......T...TGT.G.......G.....GG..........A..C...A.....AA...ATA.....A

1 P1CDO17 C......CCC.G...CTT.......C....T...........T...T.C...C......T...TGT.G.......G.....GG..........A..C...A.....AA...ATA.....A

1 P1CDO19 C......CCC.G...CTT.......C....T...........T...T.C...C......T...TGT.G.......G.....GG..........A..C...A.....AA...ATA.....A

1 P1CDO1 C......CCC.G...CTT.......C....T...........T...T.C...C......T...TGT.G.......G.....GG..........A..C...A.....AA...ATA.....A

1 P1CDO20 C......CCC.G...CTT.......C....T...........T...T.C...C......T...TGT.G.......G.....GG..........A..C...A.....AA...ATA.....A

1 P1CDO21 C......CCC.G...CTT.......C....T...........T...T.C...C......T...TGT.G.......G.....GG..........A..C...A.....AA...ATA.....A

1 P1CDO5 C......CCC.G...CTT.......C....T...........T...T.C...C......T...TGT.G.......G.....GG..........A..C...A.....AA...ATA.....A

1 P1CDO6 C......CCC.G...CTT.......C....T...........T...T.C...C......T...TGT.G.......G.....GG..........A..C...A.....AA...ATA.....A

1 P1CDO7 C......CCC.G...CTT.......C....T...........T...T.C...C......T...TGT.G.......G.....GG..........A..C...A.....AA...ATA.....A

1 P1CDO9 C......CCC.G...CTT.......C....T...........T...T.C...C......T...TGT.G.......G.....GG..........A..C...A.....AA...ATA.....A

1 P1CDO8 C......CCC.G...CTT.......C....T...........T...T.C...C......T...TGT.G.......G.....GG..........A..C...A.....AA...ATA.....A

5 P1CDO2 C......CCC.G...CTT.......C....T...........T...T.C...C......T...TGT.G.......G.....GG..........A..C...A.....AA...ATA.....A

4 P1CDO13 .........C.G.C..T.....A..C....T...........T...T...T........T....GT.G.......G.....GG..........A..C...A.....AA...ATA.....A

3 P1CDO4 C......CCC.G...CTT.......C....T...........T...T.C...C......T...TGT.G.......G...............T....C...A..A...A....A.TT...A

6 P1CDB1 (UNSWCD) C......CCC.G...CTT.......C....T...........T...T.C...C......T...TGT.G.............GG............TC......A.....T..A.....CA

19 P6UCO1 .......CCC.G...CTT....A..C..C...T..ACAA...T..TT.C.T.C..T...T.T.TGT.G.............GG.....A..T....CCA..........T.AA.TT..CA

26 13826* C.T.....C....C..T.....A..C..C..TT.........T...A...T.C......T....GT.........G........A.....................AA....A.....C.

23 H3O1 C.T....CCC.G...CT....AA..CTA.C..TA.GCAA........GC...C..T...T...............G.............G.................A....T.....CA

22 P7UCO1 .G....T..CT.....T....AA..CA..C...T.C...........G.................T......A..G.....GG........T....C...A......A...AA..G..CA

2 P1CDO3 ..T....C.....CT.......A..C...C.TT..C......T....G..T.C..T...T.......A.......G...............T....C...A..A...A....A.TT...A

2 P1CDO18 ..T....C.....CT.......A..C...C.TT..C......T....G..T.C..T...T.......A.......G...............T....C...A..A...A....A.TT...A

21 P8UCO1 ..T.....CC.G..T......AA..C...C.T...C......T...TGC.T........T....GT.........GA...........................A..A....A.....CA

17 P5CDO1 ........CC..TCT..T....A......C.T.T........T...TG..T.CT.....T....GT.........G.....GG.............C.....C......T..A...T.CA

7 P2CDO4 C......C.....C..TT....A..C..C..T.T.............G.......T...........A.......GA........................A..A..A...AA.....C.

7 P2CDO1 C......C.....C..TT....A..C..C..T.T.............G.......T...........A.......GA........................A..A..A...AA.....C.

9 P2CDO5 C......C.....C..TT....A..C..C..T.T.............G.......T...........A.......GA........................A..A..A...AA.....C.

10 P2CDO2 C.......CC...C.......AA..AA..C...T.C...........G.......T...........G.......GA........................A..A..A...AA.....C.

11 P2CDO6 C.......CC...C.......AA..AA..C...T.C...........G.......T...........G.......G.....GG.............C...A..A.....T..A.....CA

8 P2CDO3 C.......CC...C.......AA..AA..C...T.C...........G.......T...........G.......G.....GG.............C...A..A.....T..A.....CA

8 P2CDO7 C.......CC...C.......AA..AA..C...T.C...........G.......T...........G.......G.....GG.............C...A..A.....T..A.....CA

16 H5O1 ........CC...C........A..C...C.T.T.......TT...TGC.T.C...C..T....GT........T..TCTC....C....A.....C..T.....A...T.......G..

25 H4O1 .........C............A..C..C..T.T........T....GC.T.C......T....GT.G.........TCTC....CG...........AT.....A...T.......G..

24 P4CDO1 .G...A.............A..A....A...TTA......TT.........A..ATCTC.T..T.....ACG.....TCTC....C..........C..T.....A...T.......G..

15 H1O1 .G.TC.....T..C.......T.....AC....T.........GT....T.A..A.....T........ACG.....TCTC....CG...A.A......T.....A....GT.....G..

15 H1O2 .G.TC.....T..C.......T.....AC....T.........GT....T.A..A.....T........ACG.....TCTC....CG...A.A......T.....A....GT.....G..

15 H1O3 .G.TC.....T..C.......T.....AC....T.........GT....T.A..A.....T........ACG.....TCTC....CG...A.A......T.....A....GT.....G..

15 H1O4 .G.TC.....T..C.......T.....AC....T.........GT....T.A..A.....T........ACG.....TCTC....CG...A.A......T.....A....GT.....G..

15 H1O5 .G.TC.....T..C.......T.....AC....T.........GT....T.A..A.....T........ACG.....TCTC....CG...A.A......T.....A....GT.....G..

15 H1O6 .G.TC.....T..C.......T.....AC....T.........GT....T.A..A.....T........ACG.....TCTC....CG...A.A......T.....A....GT.....G..

15 H1O7 .G.TC.....T..C.......T.....AC....T.........GT....T.A..A.....T........ACG.....TCTC....CG...A.A......T.....A....GT.....G..

15 H1O8 .G.TC.....T..C.......T.....AC....T.........GT....T.A..A.....T........ACG.....TCTC....CG...A.A......T.....A....GT.....G..

15 H1O9 .G.TC.....T..C.......T.....AC....T.........GT....T.A..A.....T........ACG.....TCTC....CG...A.A......T.....A....GT.....G..

14 P3UCLW1 .G.TC...CC...C.......T.....A.....T.C.......GT......A.TA.CAC.TT.T...C.A.G.....TCTC....CG.....A.....AT.........T..........

18 H2O1 .......C.C...C....T..AATCC...C.TTACC...C..T...TG..T.CT........G.GT..G....C.G.TCT...G.CGC..AT..G...AT.....A..TT..........

13 P3UCLW2 .TT.C...............CC.....A...TTA......TT.........A..ATCTC.T.....A..........TCTC....CG............T.....A...T......AG..

12 P3UCB9 .TT.C...............CC.....A...TTA......TT.........A..ATCTC.T.....A..........TCTC....CG............T.....A...T......AG..

12 P3UCB8 .TT.C...............CC.....A...TTA......TT.........A..ATCTC.T.....A..........TCTC....CG............T.....A...T......AG..

12 P3UCB7 .TT.C...............CC.....A...TTA......TT.........A..ATCTC.T.....A..........TCTC....CG............T.....A...T......AG..

12 P3UCB6 .TT.C...............CC.....A...TTA......TT.........A..ATCTC.T.....A..........TCTC....CG............T.....A...T......AG..

12 P3UCB5 .TT.C...............CC.....A...TTA......TT.........A..ATCTC.T.....A..........TCTC....CG............T.....A...T......AG..

12 P3UCB4 .TT.C...............CC.....A...TTA......TT.........A..ATCTC.T.....A..........TCTC....CG............T.....A...T......AG..

12 P3UCB3 .TT.C...............CC.....A...TTA......TT.........A..ATCTC.T.....A..........TCTC....CG............T.....A...T......AG..

12 P3UCB2 .TT.C...............CC.....A...TTA......TT.........A..ATCTC.T.....A..........TCTC....CG............T.....A...T......AG..

12 P3UCB1 .TT.C...............CC.....A...TTA......TT.........A..ATCTC.T.....A..........TCTC....CG............T.....A...T......AG..

12 P3UCB10 .TT.C...............CC.....A...TTA......TT.........A..ATCTC.T.....A..........TCTC....CG............T.....A...T......AG..

12 P3UCO9 .TT.C...............CC.....A...TTA......TT.........A..ATCTC.T.....A..........TCTC....CG............T.....A...T......AG..

12 P3UCO8 .TT.C...............CC.....A...TTA......TT.........A..ATCTC.T.....A..........TCTC....CG............T.....A...T......AG..

12 P3UCO7 .TT.C...............CC.....A...TTA......TT.........A..ATCTC.T.....A..........TCTC....CG............T.....A...T......AG..

12 P3UCO6 .TT.C...............CC.....A...TTA......TT.........A..ATCTC.T.....A..........TCTC....CG............T.....A...T......AG..

12 P3UCO5 .TT.C...............CC.....A...TTA......TT.........A..ATCTC.T.....A..........TCTC....CG............T.....A...T......AG..

12 P3UCO4 .TT.C...............CC.....A...TTA......TT.........A..ATCTC.T.....A..........TCTC....CG............T.....A...T......AG..

12 P3UCO3 .TT.C...............CC.....A...TTA......TT.........A..ATCTC.T.....A..........TCTC....CG............T.....A...T......AG..

12 P3UCO2 .TT.C...............CC.....A...TTA......TT.........A..ATCTC.T.....A..........TCTC....CG............T.....A...T......AG..

12 P3UCO10 .TT.C...............CC.....A...TTA......TT.........A..ATCTC.T.....A..........TCTC....CG............T.....A...T......AG..

12 P3UCO1 .TT.C...............CC.....A...TTA......TT.........A..ATCTC.T.....A..........TCTC....CG............T.....A...T......AG..

20 H6O1 ...TC....C...C.......T.....AC..T.T.........GT...G..A.TA..AC.T........ACG...G...............................A....T.....CA

**MLST *aspA* | *atpA***

**type Consensus TGGATAAGAGATGTGTTTAGGGTTGAACTAATAAGATGAGGCAACATGGATGAACGCGTTAAGGTAATAGGGTTTACCATTATTCTCTCAGCCCGTTATCGTACTTCTGTCCGCTCTCTA**

1 P1CDO10 ..........GA.........A..........G.....T.......A..G...G......CT......G.......T...C.............T.......G......C.....T..C.

1 P1CDO11 ..........GA.........A..........G.....T.......A..G...G......CT......G.......T...C.............T.......G......C.....T..C.

1 P1CDO12 ..........GA.........A..........G.....T.......A..G...G......CT......G.......T...C.............T.......G......C.....T..C.

1 P1CDO14 ..........GA.........A..........G.....T.......A..G...G......CT......G.......T...C.............T.......G......C.....T..C.

1 P1CDO15 ..........GA.........A..........G.....T.......A..G...G......CT......G.......T...C.............T.......G......C.....T..C.

1 P1CDO16 ..........GA.........A..........G.....T.......A..G...G......CT......G.......T...C.............T.......G......C.....T..C.

1 P1CDO17 ..........GA.........A..........G.....T.......A..G...G......CT......G.......T...C.............T.......G......C.....T..C.

1 P1CDO19 ..........GA.........A..........G.....T.......A..G...G......CT......G.......T...C.............T.......G......C.....T..C.

1 P1CDO1 ..........GA.........A..........G.....T.......A..G...G......CT......G.......T...C.............T.......G......C.....T..C.

1 P1CDO20 ..........GA.........A..........G.....T.......A..G...G......CT......G.......T...C.............T.......G......C.....T..C.

1 P1CDO21 ..........GA.........A..........G.....T.......A..G...G......CT......G.......T...C.............T.......G......C.....T..C.

1 P1CDO5 ..........GA.........A..........G.....T.......A..G...G......CT......G.......T...C.............T.......G......C.....T..C.

1 P1CDO6 ..........GA.........A..........G.....T.......A..G...G......CT......G.......T...C.............T.......G......C.....T..C.

1 P1CDO7 ..........GA.........A..........G.....T.......A..G...G......CT......G.......T...C.............T.......G......C.....T..C.

1 P1CDO9 ..........GA.........A..........G.....T.......A..G...G......CT......G.......T...C.............T.......G......C.....T..C.

1 P1CDO8 ..........GA.........A..........G.....T.......A..G...G......CT......G.......T...C.............T.......G......C.....T..C.

5 P1CDO2 ..........GA.........A..........G.....T.......A..G...G......CT......G.......T...C.............T.......G......C.....T..C.

4 P1CDO13 ..........GA.........A..........G.....T.......A..G...G......CT......G.......T...C.............T.......G......C.....T..C.

3 P1CDO4 .....G...............A.A.......AT..........GT.A..GCA.T.....CCT...GG.G......TT...C.............T.......G......C.....T..C.

6 P1CDB1 (UNSWCD) ..A..G...............A.A.......C............T.A..GCA.T......CT...GG.G...........C....................A..C...............

19 P6UCO1 .....G...A.........A.A.A.............AG.......A.A.CA.T.........A.GG.G.A....T....C.C.....T.AT..........G......C.....T..C.

26 13826* ....................AA...........................GCA.T......CT...GG.G...C..T....C.....T...AT..A......C..C....C...TC.....

23 H3O1 .......TT.G.........AA...............A.A....T....GCA.T......CT...GG.............C......C.GA...A......C..C......T.T......

22 P7UCO1 .AA.......G.........AA........T....G.AG.....T.A.A.CA.T......CT...G..G..............C....A.AT.........A..C....CT.........

2 P1CDO3 .....G...............A.A.......AT..........GT.A..GCA.T.....CCT...GG.G......T....C.............A......A..CC...C.......T..

2 P1CDO18 .....G...............A.A.......AT..........GT.A..GCA.T.....CCT...GG.G......T....C.............A......A..CC...C.......T..

21 P8UCO1 .......TT.G.....................G.....G.....T...A.CA.T......CT...GG.............C.....T...AT.........A..C....C..........

17 P5CDO1 .....G...............A.........G............T.A...CA.T......CT...GG.G...........C.....T...AT..A......A.TC...............

7 P2CDO4 ....C.....G.........A...........G.....GA......A.A.CA.T......CT...GG.....C.......C.C.......AT.........A..C..C.....T......

7 P2CDO1 ....C.....G.........A...........G.....GA......A.A.CA.T......CT...GG.....C.......C.C.......AT.........A..C..C.....T......

9 P2CDO5 ....C.....G.........A...........G.....GA......A.A.CA.T......CT...GG.....C.......C.C.......AT.........A..C..C.....T......

10 P2CDO2 ....C.....G.........A...........G.....GA......A.A.CA.T......CT...GG.....C.......C.C......G............G......C.....T..C.

11 P2CDO6 ..A..G...............A.A.......G...........GT.A......GT.....CT...G........C.....C.C......G............G......C.....T..C.

8 P2CDO3 ..A..G...............A.A.......G...........GT.A......GT.....CT...G........C.....C.C......G............G......C.....T..C.

8 P2CDO7 ..A..G...............A.A.......G...........GT.A......GT.....CT...G........C.....C.C......G............G......C.....T..C.

16 H5O1 ...T.G..G....CAAGAG.A............TA...GA..T.T..........AAAC....A...A.A.A...G...C...........T.......T......T..C.........G

25 H4O1 ...T.G..G....CAAGAG.A............TA.....A..GA...........AAC....A...A.A.A...G.............G.TAT.CC..TT.TT............C...

24 P4CDO1 ..........G.ACAAGAG...CGCTT.GG...TA....A..T.T.......G...AAC....AA..A.A.A...G...C...........T.......T......T..C.........G

15 H1O1 ...T.G..G.....AGGAG..............TA.......T.TG.A........AAC...A....A.A.A......G....................TA.......A...A.......

15 H1O2 ...T.G..G.....AGGAG..............TA.......T.TG.A........AAC...A....A.A.A......G....................TA.......A...A.......

15 H1O3 ...T.G..G.....AGGAG..............TA.......T.TG.A........AAC...A....A.A.A......G....................TA.......A...A.......

15 H1O4 ...T.G..G.....AGGAG..............TA.......T.TG.A........AAC...A....A.A.A......G....................TA.......A...A.......

15 H1O5 ...T.G..G.....AGGAG..............TA.......T.TG.A........AAC...A....A.A.A......G....................TA.......A...A.......

15 H1O6 ...T.G..G.....AGGAG..............TA.......T.TG.A........AAC...A....A.A.A......G....................TA.......A...A.......

15 H1O7 ...T.G..G.....AGGAG..............TA.......T.TG.A........AAC...A....A.A.A......G....................TA.......A...A.......

15 H1O8 ...T.G..G.....AGGAG..............TA.......T.TG.A........AAC...A....A.A.A......G....................TA.......A...A.......

15 H1O9 ...T.G..G.....AGGAG..............TA.......T.TG.A........AAC...A....A.A.A......G....................TA.......A...A.......

14 P3UCLW1 A..T.G..G.....AAGAG........G.....TACA...CA...G.A........AAC....A...A.A.A.A.G.T.C.G.CTA........A..GCTA..TC.T..C........C.

18 H2O1 ...C.GG..A..ACAAGAG.AA...........TA...GA...GT...........AAC....A.G.A.A.A...G.............G.TAT.CC..TT.TT............C...

13 P3UCLW2 ..AC....GA...CAAGAG.A.........T..TA.....A..GA...........AAC....A...A.A.A...G.............G.TAT.CC..TT.TT............C...

12 P3UCB9 ..AC....GA...CAAGAG.A.........T..TA.....A..GA...........AAC....A...A.A.A...G.............G.TAT.CC..TT.TT............C...

12 P3UCB8 ..AC....GA...CAAGAG.A.........T..TA.....A..GA...........AAC....A...A.A.A...G.............G.TAT.CC..TT.TT............C...

12 P3UCB7 ..AC....GA...CAAGAG.A.........T..TA.....A..GA...........AAC....A...A.A.A...G.............G.TAT.CC..TT.TT............C...

12 P3UCB6 ..AC....GA...CAAGAG.A.........T..TA.....A..GA...........AAC....A...A.A.A...G.............G.TAT.CC..TT.TT............C...

12 P3UCB5 ..AC....GA...CAAGAG.A.........T..TA.....A..GA...........AAC....A...A.A.A...G.............G.TAT.CC..TT.TT............C...

12 P3UCB4 ..AC....GA...CAAGAG.A.........T..TA.....A..GA...........AAC....A...A.A.A...G.............G.TAT.CC..TT.TT............C...

12 P3UCB3 ..AC....GA...CAAGAG.A.........T..TA.....A..GA...........AAC....A...A.A.A...G.............G.TAT.CC..TT.TT............C...

12 P3UCB2 ..AC....GA...CAAGAG.A.........T..TA.....A..GA...........AAC....A...A.A.A...G.............G.TAT.CC..TT.TT............C...

12 P3UCB1 ..AC....GA...CAAGAG.A.........T..TA.....A..GA...........AAC....A...A.A.A...G.............G.TAT.CC..TT.TT............C...

12 P3UCB10 ..AC....GA...CAAGAG.A.........T..TA.....A..GA...........AAC....A...A.A.A...G.............G.TAT.CC..TT.TT............C...

12 P3UCO9 ..AC....GA...CAAGAG.A.........T..TA.....A..GA...........AAC....A...A.A.A...G.............G.TAT.CC..TT.TT............C...

12 P3UCO8 ..AC....GA...CAAGAG.A.........T..TA.....A..GA...........AAC....A...A.A.A...G.............G.TAT.CC..TT.TT............C...

12 P3UCO7 ..AC....GA...CAAGAG.A.........T..TA.....A..GA...........AAC....A...A.A.A...G.............G.TAT.CC..TT.TT............C...

12 P3UCO6 ..AC....GA...CAAGAG.A.........T..TA.....A..GA...........AAC....A...A.A.A...G.............G.TAT.CC..TT.TT............C...

12 P3UCO5 ..AC....GA...CAAGAG.A.........T..TA.....A..GA...........AAC....A...A.A.A...G.............G.TAT.CC..TT.TT............C...

12 P3UCO4 ..AC....GA...CAAGAG.A.........T..TA.....A..GA...........AAC....A...A.A.A...G.............G.TAT.CC..TT.TT............C...

12 P3UCO3 ..AC....GA...CAAGAG.A.........T..TA.....A..GA...........AAC....A...A.A.A...G.............G.TAT.CC..TT.TT............C...

12 P3UCO2 ..AC....GA...CAAGAG.A.........T..TA.....A..GA...........AAC....A...A.A.A...G.............G.TAT.CC..TT.TT............C...

12 P3UCO10 ..AC....GA...CAAGAG.A.........T..TA.....A..GA...........AAC....A...A.A.A...G.............G.TAT.CC..TT.TT............C...

12 P3UCO1 ..AC....GA...CAAGAG.A.........T..TA.....A..GA...........AAC....A...A.A.A...G.............G.TAT.CC..TT.TT............C...

20 H6O1 .......TT.G.........AA...............A.A....T....GCA.T......CT...GG.......................AT.........A..C....C..........

**MLST *atpA* | *glnA* | *pgi***

**type Consensus ACTACCCCAAAGCGTCTCCACCTCCCATGATTAAACCACATCGCTCGTTCCTCCCCCCCGAAGTTCCCCCGTACTCCCGAACCATCTAGCCCCCCTACCAGTGAGGACAAACTCTTCCTC**

1 P1CDO10 GT............A....G......G.A........G.....................A........T..C...TTT...................T......................

1 P1CDO11 GT............A....G......G.A........G.....................A........T..C...TTT...................T......................

1 P1CDO12 GT............A....G......G.A........G.....................A........T..C...TTT...................T......................

1 P1CDO14 GT............A....G......G.A........G.....................A........T..C...TTT...................T......................

1 P1CDO15 GT............A....G......G.A........G.....................A........T..C...TTT...................T......................

1 P1CDO16 GT............A....G......G.A........G.....................A........T..C...TTT...................T......................

1 P1CDO17 GT............A....G......G.A........G.....................A........T..C...TTT...................T......................

1 P1CDO19 GT............A....G......G.A........G.....................A........T..C...TTT...................T......................

1 P1CDO1 GT............A....G......G.A........G.....................A........T..C...TTT...................T......................

1 P1CDO20 GT............A....G......G.A........G.....................A........T..C...TTT...................T......................

1 P1CDO21 GT............A....G......G.A........G.....................A........T..C...TTT...................T......................

1 P1CDO5 GT............A....G......G.A........G.....................A........T..C...TTT...................T......................

1 P1CDO6 GT............A....G......G.A........G.....................A........T..C...TTT...................T......................

1 P1CDO7 GT............A....G......G.A........G.....................A........T..C...TTT...................T......................

1 P1CDO9 GT............A....G......G.A........G.....................A........T..C...TTT...................T......................

1 P1CDO8 GT............A....G......G.A........G.....................A........T..C...TTT...................T......................

5 P1CDO2 GT............A....G......G.A........G.....................A........T..C...TTT...................T......................

4 P1CDO13 GT............A....G......G.A........G.....................A........T..C...TTT...................T......................

3 P1CDO4 GT............A....G......G.A........G.....................A........T..C...TTT...................T......................

6 P1CDB1 (UNSWCD) .TC.T.........GT......C.....A..C.....G....A.C..............................T........C......T.............CT........C....

19 P6UCO1 ..............G........T....A..............T.A...........T................C............TA...........A.............CC....

26 13826* .TCGT.........A....G..C.....AG.G.G....T..T.T.............................T.T........C..T...........................C...T

23 H3O1 .TC.T.........A.......C...GCAG.C...........................................T................T...........A..........C....

22 P7UCO1 .TC.T.........A.............AG.C..........................TA...............T.......................................C....

2 P1CDO3 .TC.T.T.......G............C.........................T....................C.T...........AT..........A...................

2 P1CDO18 .TC.T.T.......G............C.........................T....................C.T...........AT..........A...................

21 P8UCO1 .TC.T.........AT......C...G.AG......TG...........................T.........TTT..........A..T............A.........CC....

17 P5CDO1 .TC.T.........G.......C...G.AG.C.....G.................T...................T............................................

7 P2CDO4 .TC.T.........A.......C.....A........G.......T......................................................A..G................

7 P2CDO1 .TC.T.........A.......C.....A........G.......T......................................................A..G................

9 P2CDO5 .TC.T.........A.......C.....A........G.......T......................................................A..G................

10 P2CDO2 ........G.....A.........T...AG............................................C............TA..A.....T..AC..A..........CT...

11 P2CDO6 ........G.....A.........T...AG............................................C............TA..A.....T..AC..A..........CT...

8 P2CDO3 ........G.....A.........T...AG............................................C............TA..A.....T..AC..A..........CT...

8 P2CDO7 ........G.....A.........T...AG............................................C............TA..A.....T..AC..A..........CT...

16 H5O1 ...G.....TG...A.............A..............................................T............A..........................C....

25 H4O1 ..C......T..T.G.....T.C...GC...C..............................ACC....T....C.............................A...............

24 P4CDO1 ...G.....TGATA..CTT........C...C.GCT...TAT....A.CTT.TT..G.....A...TA.....T.T.........TC.ATTT.T..C.TT.......T.......C....

15 H1O1 ...G.T.T....TA....T..T...A.C..C.C......TATAT..ACCTTCT.........A....A...CG...TTTGGATGC..G.T..T..C..TT..A..A...CGTA..AT...

15 H1O2 ...G.T.T....TA....T..T...A.C..C.C......TATAT..ACCTTCT.........A....A...CG...TTTGGATGC..G.T..T..C..TT..A..A...CGTA..AT...

15 H1O3 ...G.T.T....TA....T..T...A.C..C.C......TATAT..ACCTTCT.........A....A...CG...TTTGGATGC..G.T..T..C..TT..A..A...CGTA..AT...

15 H1O4 ...G.T.T....TA....T..T...A.C..C.C......TATAT..ACCTTCT.........A....A...CG...TTTGGATGC..G.T..T..C..TT..A..A...CGTA..AT...

15 H1O5 ...G.T.T....TA....T..T...A.C..C.C......TATAT..ACCTTCT.........A....A...CG...TTTGGATGC..G.T..T..C..TT..A..A...CGTA..AT...

15 H1O6 ...G.T.T....TA....T..T...A.C..C.C......TATAT..ACCTTCT.........A....A...CG...TTTGGATGC..G.T..T..C..TT..A..A...CGTA..AT...

15 H1O7 ...G.T.T....TA....T..T...A.C..C.C......TATAT..ACCTTCT.........A....A...CG...TTTGGATGC..G.T..T..C..TT..A..A...CGTA..AT...

15 H1O8 ...G.T.T....TA....T..T...A.C..C.C......TATAT..ACCTTCT.........A....A...CG...TTTGGATGC..G.T..T..C..TT..A..A...CGTA..AT...

15 H1O9 ...G.T.T....TA....T..T...A.C..C.C......TATAT..ACCTTCT.........A....A...CG...TTTGGATGC..G.T..T..C..TT..A..A...CGTA..AT...

14 P3UCLW1 ...........ATA..C.T.T........G.............T..A.CTTATTA.G..ATGA.C.TT..AC....TT.GGATGC..G.T..T.TC..T..C..A...G......CT...

18 H2O1 ..C...T..T.ATA..C.T.T........G.............T..A.CTTATTA.G..ATGA.C.TT..AC....TT.GGATGC..G.T..T.TC..T......A..............

13 P3UCLW2 ..C...T..T..TA....T......A.C..C.C............................................................................CG.ATC.....

12 P3UCB9 ..C...T..T..TA....T......A.C..C.C............................................................................CG.ATC.....

12 P3UCB8 ..C...T..T..TA....T......A.C..C.C............................................................................CG.ATC.....

12 P3UCB7 ..C...T..T..TA....T......A.C..C.C............................................................................CG.ATC.....

12 P3UCB6 ..C...T..T..TA....T......A.C..C.C............................................................................CG.ATC.....

12 P3UCB5 ..C...T..T..TA....T......A.C..C.C............................................................................CG.ATC.....

12 P3UCB4 ..C...T..T..TA....T......A.C..C.C............................................................................CG.ATC.....

12 P3UCB3 ..C...T..T..TA....T......A.C..C.C............................................................................CG.ATC.....

12 P3UCB2 ..C...T..T..TA....T......A.C..C.C............................................................................CG.ATC.....

12 P3UCB1 ..C...T..T..TA....T......A.C..C.C............................................................................CG.ATC.....

12 P3UCB10 ..C...T..T..TA....T......A.C..C.C............................................................................CG.ATC.....

12 P3UCO9 ..C...T..T..TA....T......A.C..C.C............................................................................CG.ATC.....

12 P3UCO8 ..C...T..T..TA....T......A.C..C.C............................................................................CG.ATC.....

12 P3UCO7 ..C...T..T..TA....T......A.C..C.C............................................................................CG.ATC.....

12 P3UCO6 ..C...T..T..TA....T......A.C..C.C............................................................................CG.ATC.....

12 P3UCO5 ..C...T..T..TA....T......A.C..C.C............................................................................CG.ATC.....

12 P3UCO4 ..C...T..T..TA....T......A.C..C.C............................................................................CG.ATC.....

12 P3UCO3 ..C...T..T..TA....T......A.C..C.C............................................................................CG.ATC.....

12 P3UCO2 ..C...T..T..TA....T......A.C..C.C............................................................................CG.ATC.....

12 P3UCO10 ..C...T..T..TA....T......A.C..C.C............................................................................CG.ATC.....

12 P3UCO1 ..C...T..T..TA....T......A.C..C.C............................................................................CG.ATC.....

20 H6O1 .TC.T......ATA..C.T.T........G.............T..A.CTTATTA.G..ATGA.C.TT..AC....TT.GGA.GC..G.T..T.TC..T.......T.....A.CC.TC.

**MLST *pgi* | *tkt***

**type Consensus TGCAGTCAGGATCCCCCAGGAGCATGCAGACCGCTCGCTACCCATATTGGCGCGGGCATCCTTCGCCGACATCGGGCGGACGGCCTGCAAGGGGGGCGAGCTGCTGAGGGCGCTAGAAGA**

1 P1CDO10 C...AA................GTA.................A.............A......T............T...........................................

1 P1CDO11 C...AA................GTA.................A.............A......T............T...........................................

1 P1CDO12 C...AA................GTA.................A.............A......T............T...........................................

1 P1CDO14 C...AA................GTA.................A.............A......T............T...........................................

1 P1CDO15 C...AA................GTA.................A.............A......T............T...........................................

1 P1CDO16 C...AA................GTA.................A.............A......T............T...........................................

1 P1CDO17 C...AA................GTA.................A.............A......T............T...........................................

1 P1CDO19 C...AA................GTA.................A.............A......T............T...........................................

1 P1CDO1 C...AA................GTA.................A.............A......T............T...........................................

1 P1CDO20 C...AA................GTA.................A.............A......T............T...........................................

1 P1CDO21 C...AA................GTA.................A.............A......T............T...........................................

1 P1CDO5 C...AA................GTA.................A.............A......T............T...........................................

1 P1CDO6 C...AA................GTA.................A.............A......T............T...........................................

1 P1CDO7 C...AA................GTA.................A.............A......T............T...........................................

1 P1CDO9 C...AA................GTA.................A.............A......T............T...........................................

1 P1CDO8 C...AA................GTA.................A.............A......T............T...........................................

5 P1CDO2 C...AA................GTA.................A.............A......T............T...........................................

4 P1CDO1 3 C...AA................GTA.................A.............A......T............T...........................................

3 P1CDO4 C...AA................GTA.................A.............A......T............T...........................................

6 P1CDB1 (UNSWCD) ....A......CT.TT...AG.......A.TT..........A....A.C.T....A......T............T............GA..AT...G.A..T.A..AAT...G.T...

19 P6UCO1 ..T..A...........G.AG...A............A....ACA...........T.CT................T..G.........G.A..A...G.A...............C..G

26 13826* .....A..A......T......GTA..GA..T........T.........T...ACT..T.C.A............T...........................................

23 H3O1 ....AA.............AG...A.................A.............T..............CT.....................A..AG.A.....G.......G.....

22 P7UCO1 .....A.............AG...A.................ACA...........T..................A.A..T.........A....A..G.A.A.....A...........

2 P1CDO3 ....AA....G........AG...A.T.A.T...C.A..........A.C.....CT.......A.....GC.....A..TT..T.....AA..A...G.A..T............C...

2 P1CDO18 ....AA....G........AG...A.T.A.T...C.A..........A.C.....CT.......A.....GC.....A..TT..T.....AA..A...G.A..T............C...

21 P8UCO1 ........T............TGGA.................A.............T.............GC.....A..TT..T.....AA..A...G.A..T............C...

17 P5CDO1 .....A...........G.AG...A.................A.............A.......AATTTTGC...A..T...T...A.......A...G.A.....G......GG.....

7 P2CDO4 .....A..ATG........AG......CA...........T..............CT........A....GC..................A.A.A....TA.....G.......G.....

7 P2CDO1 .....A..ATG........AG......CA...........T..............CT........A....GC..................A.A.A....TA.....G.......G.....

9 P2CDO5 .....A..ATG........AG......CA...........T..............CT........ATTT.GC..A............TG...A.C..AG.A..T........T.G..TA.

10 P2CDO2 ...GA...A....T........G..A.G........A.............T........T.CG..ATTT.GC..A............TG...A.C..AG.A..T........T.G..TA.

11 P2CDO6 ...GA...A....T........G..A.G........A.............T........T.CG..A....GC..................A.A.A....TA.....G.......G.....

8 P2CDO3 ...GA...A....T........G..A.G........A.............T........T.CG..ATTT.GC..A............TG...A.C..AG.A..T........T.G..TA.

8 P2CDO7 ...GA...A....T........G..A.G........A.............T........T.CG..ATTT.GC..A............TG...A.C..AG.A..T........T.G..TA.

16 H5O1 ....AA.............A....A.................A.............T..T................T..G.........G.A..A..AG.A...............C...

25 H4O1 .....A.............AG...A..............G..A.............T.............G...................A......AG.A...............C...

24 P4CDO1 ....AA...........GAAG...A........T........A.......T..A..T.CT..............A...........A..G.A............C......A........

15 H1O1 .A.............TA.....A....G.......TA.........C.....T..T...T.CGT..T...GC...A....TT.G......A....TAAG.A.A....A.......A.TAG

15 H1O2 .A.............TA.....A....G.......TA.........C.....T..T...T.CGT..T...GC...A....TT.G......A....TAAG.A.A....A.......A.TAG

15 H1O3 .A.............TA.....A....G.......TA.........C.....T..T...T.CGT..T...GC...A....TT.G......A....TAAG.A.A....A.......A.TAG

15 H1O4 .A.............TA.....A....G.......TA.........C.....T..T...T.CGT..T...GC...A....TT.G......A....TAAG.A.A....A.......A.TAG

15 H1O5 .A.............TA.....A....G.......TA.........C.....T..T...T.CGT..T...GC...A....TT.G......A....TAAG.A.A....A.......A.TAG

15 H1O6 .A.............TA.....A....G.......TA.........C.....T..T...T.CGT..T...GC...A....TT.G......A....TAAG.A.A....A.......A.TAG

15 H1O7 .A.............TA.....A....G.......TA.........C.....T..T...T.CGT..T...GC...A....TT.G......A....TAAG.A.A....A.......A.TAG

15 H1O8 .A.............TA.....A....G.......TA.........C.....T..T...T.CGT..T...GC...A....TT.G......A....TAAG.A.A....A.......A.TAG

15 H1O9 .A.............TA.....A....G.......TA.........C.....T..T...T.CGT..T...GC...A....TT.G......A....TAAG.A.A....A.......A.TAG

14 P3UCLW1 .....A....G....T......G....G...............CA.....T....CT..T..............A..............G.A............C......A........

18 H2O1 ......T.A.G...........G....G.......TA.....ACC...A.T....C........T.........A..............G.A............C......A........

13 P3UCLW2 ..........G.T.............T.......C......A...G....T......G....G...........A..............G.A............C......A........

12 P3UCB9 ..........G.T.............T..T....C......A...G....T......G....G...........A..............G.A............C......A........

12 P3UCB8 ..........G.T.............T..T....C......A...G....T......G....G...........A..............G.A............C......A........

12 P3UCB7 ..........G.T.............T..T....C......A...G....T......G....G...........A..............G.A............C......A........

12 P3UCB6 ..........G.T.............T..T....C......A...G....T......G....G...........A..............G.A............C......A........

12 P3UCB5 ..........G.T.............T..T....C......A...G....T......G....G...........A..............G.A............C......A........

12 P3UCB4 ..........G.T.............T..T....C......A...G....T......G....G...........A..............G.A............C......A........

12 P3UCB3 ..........G.T.............T..T....C......A...G....T......G....G...........A..............G.A............C......A........

12 P3UCB2 ..........G.T.............T..T....C......A...G....T......G....G...........A..............G.A............C......A........

12 P3UCB1 ..........G.T.............T..T....C......A...G....T......G....G...........A..............G.A............C......A........

12 P3UCB10 ..........G.T.............T..T....C......A...G....T......G....G...........A..............G.A............C......A........

12 P3UCO9 ..........G.T.............T..T....C......A...G....T......G....G...........A..............G.A............C......A........

12 P3UCO8 ..........G.T.............T..T....C......A...G....T......G....G...........A..............G.A............C......A........

12 P3UCO7 ..........G.T.............T..T....C......A...G....T......G....G...........A..............G.A............C......A........

12 P3UCO6 ..........G.T.............T..T....C......A...G....T......G....G...........A..............G.A............C......A........

12 P3UCO5 ..........G.T.............T..T....C......A...G....T......G....G...........A..............G.A............C......A........

12 P3UCO4 ..........G.T.............T..T....C......A...G....T......G....G...........A..............G.A............C......A........

12 P3UCO3 ..........G.T.............T..T....C......A...G....T......G....G...........A..............G.A............C......A........

12 P3UCO2 ..........G.T.............T..T....C......A...G....T......G....G...........A..............G.A............C......A........

12 P3UCO10 ..........G.T.............T..T....C......A...G....T......G....G...........A..............G.A............C......A........

12 P3UCO1 ..........G.T.............T..T....C......A...G....T......G....G...........A..............G.A............C......A........

20 H6O1 ....AA.TA.G.T......AG...A.......T.C...A...A.............T...T...A....T...T.A.........G.....AA.....G.AC..C..............G

**MLST *tkt* |**

**type Consensus TAAAGTAGCAAGGCTCATATGAATGGTAT**

1 P1CDO10 .G..A....G.A.........G.......

1 P1CDO11 .G..A....G.A.........G.......

1 P1CDO12 .G..A....G.A.........G.......

1 P1CDO14 .G..A....G.A.........G.......

1 P1CDO15 .G..A....G.A.........G.......

1 P1CDO16 .G..A....G.A.........G.......

1 P1CDO17 .G..A....G.A.........G.......

1 P1CDO19 .G..A....G.A.........G.......

1 P1CDO1 .G..A....G.A.........G.......

1 P1CDO20 .G..A....G.A.........G.......

1 P1CDO21 .G..A....G.A.........G.......

1 P1CDO5 .G..A....G.A.........G.......

1 P1CDO6 .G..A....G.A.........G.......

1 P1CDO7 .G..A....G.A.........G.......

1 P1CDO9 .G..A....G.A.........G.......

1 P1CDO8 .G..A....G.A.........G.......

5 P1CDO2 .G..A...TG.A.........G.......

4 P1CDO13 .G..A....G.A.........G.......

3 P1CDO4 .G..A...TG.A.........G.......

6 P1CDB1 (UNSWCD) ...G.........................

19 P6UCO1 AG.GA.....GA.................

26 13826* .G..A....G.A.................

23 H3O1 ...G..T..GGA.................

22 P7UCO1 AG..........A.....G..........

2 P1CDO3 ..GG.........................

2 P1CDO18 ..GG.........................

21 P8UCO1 ..GG...................G.A.GC

17 P5CDO1 ......C.....A...G..G..T.A..G.

7 P2CDO4 AG.........A...........G.A.GC

7 P2CDO1 AG.........A...........G.A.GC

9 P2CDO5 AG.........A.......G.GTC...G.

10 P2CDO2 AG.........A.......G.GTC...G.

11 P2CDO6 AG.........A...........G.A.GC

8 P2CDO3 AG.........A.......G.GTC...G.

8 P2CDO7 AG.........A.......G.GTC...G.

16 H5O1 .G.G..G...GA..CT..G..........

25 H4O1 .G.G..G.......CT..G..........

24 P4CDO1 .......T.....................

15 H1O1 A..GT............AG.A..C...G.

15 H1O2 A..GT............AG.A..C...G.

15 H1O3 A..GT............AG.A..C...G.

15 H1O4 A..GT............AG.A..C...G.

15 H1O5 A..GT............AG.A..C...G.

15 H1O6 A..GT............AG.A..C...G.

15 H1O7 A..GT............AG.A..C...G.

15 H1O8 A..GT............AG.A..C...G.

15 H1O9 A..GT............AG.A..C...G.

14 P3UCLW1 .............................

18 H2O1 .............................

13 P3UCLW2 .............................

12 P3UCB9 .............................

12 P3UCB8 .............................

12 P3UCB7 .............................

12 P3UCB6 .............................

12 P3UCB5 .............................

12 P3UCB4 .............................

12 P3UCB3 .............................

12 P3UCB2 .............................

12 P3UCB1 .............................

12 P3UCB10 .............................

12 P3UCO9 .............................

12 P3UCO8 .............................

12 P3UCO7 .............................

12 P3UCO6 .............................

12 P3UCO5 .............................

12 P3UCO4 .............................

12 P3UCO3 .............................

12 P3UCO2 .............................

12 P3UCO10 .............................

12 P3UCO1 .............................

20 H6O1 .....G..T....G...AG....C..A..

**Campylobacter concisus* strain 13826 (Accession No.: CP000792.1)
